# Correlation of Optical Coherence Tomography Angiography Characteristics with Visual Function to Define Vision-Threatening Diabetic Macular Ischemia

**DOI:** 10.3390/diagnostics12051050

**Published:** 2022-04-22

**Authors:** Wei-Shan Tsai, Sridevi Thottarath, Sarega Gurudas, Piyali Sen, Elizabeth Pearce, Andrea Giani, Victor Chong, Chui Ming Gemmy Cheung, Sobha Sivaprasad

**Affiliations:** 1 NIHR Moorfields Biomedical Research Centre, Moorfields Eye Hospital NHS Foundation Trust, 162 City Road, London EC1V 2PD, UK; wei-shan.tsai@nhs.net (W.-S.T.); s.thottarath@nhs.net (S.T.); 2 Institute of Ophthalmology, University College London, 11-43 Bath Street, London EC1V 9EL, UK; sarega.g@gmail.com (S.G.); p.sen@nhs.net (P.S.); victor@eretina.org (V.C.); 3 Boehringer Ingelheim, Binger Street 173, 55218 Ingelheim am Rhein, Germany; liz.pearce@boehringer-ingelheim.com (E.P.); andrea.giani@boehringer-ingelheim.com (A.G.); 4 Singapore National Eye Centre, 11 Third Hospital Avenue, Singapore 168751, Singapore; gemmy.cheung.c.m@singhealth.com.sg

**Keywords:** diabetic macular ischemia, optical coherence tomography angiography, visual acuity, low-luminance visual acuity, proliferative diabetic retinopathy, panretinal photocoagulation

## Abstract

The thresholds of macular microvasculature parameters associated with mild visual impairment in diabetic macular ischemia (DMI) patients are unclear. Therefore, this prospective observational study is aimed at demonstrating the optical coherence tomography angiography parameters that best correlate with mild visual impairment (<70 Early Treatment Diabetic Retinopathy Study (ETDRS) letters, Snellen equivalent 20/40) in DMI. The study was completed at the Moorfields Eye Hospital from December 2019 to August 2021. A total of 123 eyes of 87 patients with stable-treated proliferative diabetic retinopathy following panretinal photocoagulation were recruited. DMI was defined as an irregular foveal avascular zone (FAZ) area ≥ 0.5 mm^2^ or a smaller FAZ area with parafoveal capillary dropout in at least one quadrant. The analysis showed that the whole image deep vascular complex vessel density (DVC VD) in the 3 × 3 mm area had the best discriminatory ability to identify participants with mild visual impairment at 41.9% (area under the curve = 0.77, sensitivity 94%, specificity 54%, likelihood ratio [LR] = 2.04), and the FAZ area had the greatest post-test LR = 4.21 at 0.64 mm^2^. The 3 × 3 mm whole image DVC VD and FAZ area cutoffs are useful for screening vision-threatening DMI, but DVC VD has low specificity.

## 1. Introduction

Diabetic macular ischemia (DMI) is traditionally defined based on the fluorescein angiographic (FA) evidence of an enlarged and irregular foveal avascular zone (FAZ) area of at least 0.5 mm^2^ or parafoveal capillary dropout in at least one parafoveal quadrant if the FAZ area is less than 0.5 mm^2^ [1]. However, these FAZ parameters are not always associated with visual impairment [2]. It is useful to explore the anatomical-based parameters that best correlate with early visual loss.

FA is a well-established investigation but only provides information on the integrity of superficial vascular complex (SVC) [3]. With the advent of optical coherence tomography angiography (OCTA), it is now possible to non-invasively visualize and quantify the SVC and other FAZ parameters [4]. Moreover, the intermediate capillary plexus (ICP) and deep capillary plexus (DCP), together termed the deep vascular complex (DVC), are also readily visible for analysis [5]. Furthermore, the microvasculature images in the macula are more clearly outlined as OCTA utilizes an infrared light source that is not absorbed by the macular xanthophyll pigment [6]. The correlations of these vascular parameters with early visual function losses in DMI may help us define vision-threatening DMI.

Eyes with proliferative diabetic retinopathy (PDR) are more prone to have co-existing DMI [2]. Therefore, studying DMI characteristics in PDR eyes is more efficient and allows for homogeneity of the study cohort. However, not all eyes with PDR have an enlarged FAZ [7]. Most importantly, these eyes have significant inter-individual differences in visual acuity outcomes despite attaining stability following panretinal photocoagulation [8]. Changes in vascular parameters at the macula could explain this variability. In addition, as diabetic macular edema (DME) is the most common cause of mild visual impairment in these eyes, excluding eyes with DME will provide more specific information on DMI.

This study aimed to determine the correlation of macular microvascular parameters measured on OCTA with visual function in DMI to evaluate the microvasculature changes associated mild visual impairment. Best-corrected visual acuity (BCVA) is a foveal cone function [9]; however, DMI also affects the parafoveal capillary density [10], which influences rod photoreceptors. As low-luminance visual acuity (LLVA) is dependent on an intact transduction pathway involving both rods and cones [11], we speculated that LLVA might correlate better with DMI. We have previously reported that BCVA correlated well with LLVA only at higher levels of BCVA in PDR [12]. Therefore, correlating these visual function measures with vascular parameters in the eyes with DMI may identify the threshold of these parameters that are best associated with mild visual impairment.

## 2. Materials and Methods

### 2.1. Study Design

This prospective cross-sectional study followed the tenets of the Declaration of Helsinki and was approved by the United Kingdom (UK) National Research Ethics Committee Service (19/NI/0030). Written informed consent was obtained.

### 2.2. Participants

Patients with stable-treated PDR, defined as no active neovascularization for at least six months following panretinal photocoagulation in the study eye, were identified from the retinal clinics at the Moorfields Eye Hospital from December 2019 to August 2021. Patients were recruited only if the FAZ area on OCTA was at least 0.5 mm^2^ or parafoveal capillary dropout was present in at least one parafoveal quadrant if the FAZ area was less than 0.5 mm^2^. In addition, only eyes with normal to moderate visual impairment were included, defined as BCVA of at least 40 Early Treatment Diabetic Retinopathy Study (ETDRS) letters (Snellen equivalent 20/160). Both eyes were included if they met the eligibility criteria.

Key exclusion criteria were eyes with optical coherence tomography (OCT) evidence of DME, history of intravitreal injections in the last six months, a visually disabling cataract likely to be decreasing the BCVA by three lines or reducing BCVA to 20/40 or worse, or any ocular condition that, in the view of the investigator, was likely to affect the visual function of the study eye.

### 2.3. Visual Function Assessment

Refraction and BCVA were assessed by masked optometrists using a high-contrast ETDRS chart (Precision Vision, Bloomington, IL, USA) at 4 m. The LLVA was measured by placing a neutral density filter in front of the ETDRS chart, reducing the luminance by 2.0 log units without interfering with the room lighting conditions [12]. The total ETDRS letters that the patient could read with each eye were recorded for both BCVA and LLVA.

### 2.4. OCTA Acquisition

**Figure 1:**
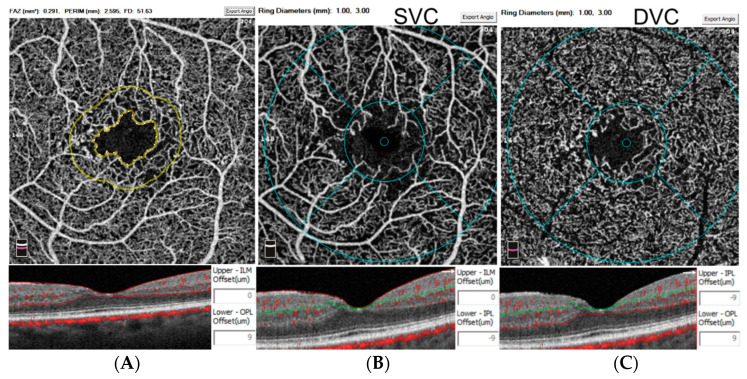
Overview of the measurements on optical coherence tomography angiography. The foveal avascular zone (FAZ) area, perimeter, and parafoveal 300-µm ring vessel density (FD–300) were automatically measured in the slab between the internal limiting membrane (ILM) and 9 µm below the outer plexiform layer (OPL) (**A**). The whole image and parafoveal vessel density were quantified in the superficial vascular complex (SVC) between the ILM and 9 µm above the inner plexiform layer (IPL) (**B**) and the deep vascular complex (DVC) between 9 µm above the IPL and 9 µm below the OPL (**C**).

A commercial spectral-domain OCTA (Avanti RTVUE-XR; Optovue, Fremont, CA, USA, version 2018.0.0.18) was employed to acquire OCTA images [13]. Foveal-centered 3 × 3 mm scans of the macular area were obtained from each eye. The split-spectrum amplitude-decorrelation angiography (SSADA) in the Optovue system was utilized to generate the perfusion maps [14]. After acquiring images, the retinal layers were automatically segmented using the inbuilt Optovue software (Figure 1). The SVC slab was defined as the space between the internal limiting membrane (ILM) and 9 µm above the inner plexiform layer (IPL). The DVC window was referred to the portion containing 9 µm above the IPL to 9 µm below the outer plexiform layer (OPL) [15].

An inbuilt software monitored the quality of the OCTA images, and all the images with a quality score of less than five were excluded. Two examiners then screened for possible artifacts separately and disqualified those with evident blink artifacts from entering the database. Segmentation errors were manually corrected if necessary [16]. The device’s inbuilt algorithm rectified the projection and motion artifacts [17,18].

In the FAZ mode, the software automatically delineated and calculated the FAZ perimeter and area in the SVC. Manual adjustments were made if any vessel was erroneously included inside the FAZ or the device misinterpreted the suspended scattering particles in motion (SSPiM) as part of the FAZ margin [19]. The FAZ acircularity index (FAZ–AI) was also calculated as the ratio of the studied perimeter to the circumference of a reference circle that had the same area as the studied FAZ [20]. The parafoveal 300-µm ring vessel density (FD–300), representing the capillary vessel density in the 300-µm outskirts of the FAZ [21], was also recorded.

The superficial vascular complex vessel density (SVC VD) and deep vascular complex vessel density (DVC VD) in the 3 × 3 mm whole image and parafoveal region (the annular area between 1 to 3 mm-diameter circle) were automatically analyzed in the density mode.

### 2.5. Statistical Analysis

The categorical parameters are reported as a number or a percentage, while the continuous variables are recorded using mean and standard deviation. The ETDRS letter scores were used for statistical analysis without converting to the logarithm of the minimum angle of resolution (logMAR) visual acuity (VA). As data from both eyes of some subjects were available, generalized estimating equations (GEE) assuming an unstructured correlation structure and robust standard errors were used to test univariate differences between the two sets of data (binary dependent variable) while accounting for inter-eye correlation. The statistical significance was defined as a *p*-value less than 0.05. The Pearson coefficients (*r*) were calculated to measure the correlation between two variables. A Pearson correlation coefficient of 0.80 to 1.00, 0.60 to 0.79, 0.40 to 0.59, 0.20 to 0.39 and 0.00 to 0.19 was considered a very strong, strong, moderate, weak, and very weak correlation, respectively. Receiver operating characteristic (ROC) curves were plotted, and clustered bootstrapping with 1000 replicates was used to estimate the bias-corrected 95% confidence interval (95% CI) for the area under the curve (AUC) [22,23,24]. Bootstrapping allows for probability-based inference for the AUC and corrects for the inter-eye correlation with respect to the estimation of the standard error of the estimated AUC. An AUC of 0.7 or more was considered very good, whereas an AUC between 0.6 and 0.7 was considered acceptable [22]. The cutoff point was appointed to a specific number with the largest sum of sensitivity and specificity. The 70 ETDRS letters VA (Snellen equivalent 20/40) was chosen as this cutoff is conventionally used as the upper limit of mild visual impairment in retinal clinical trials. The cutoff for LLVA was also chosen as 70 letters based on our previous study, which showed that approximately 50% of the PDR patients had 70 letters or better in the CLARITY trial [12]. Statistical analysis was performed using the Stata MP version 15 [25].

## 3. Results

### 3.1. Demographics and Clinical Characteristics

A total of 87 patients were recruited for the study. Among all, 123 eyes were eligible. The main reasons for excluding the 51 eyes were co-existing DME, visually disabling epiretinal membrane, and active PDR. All studied eyes had a spherical equivalent (SE) of +/− 6 diopters (D) or less, except one with high myopia (SE = −13.125 D). Table 1 shows the demographics and disease characteristics of the study participants.

**Figure 2:**
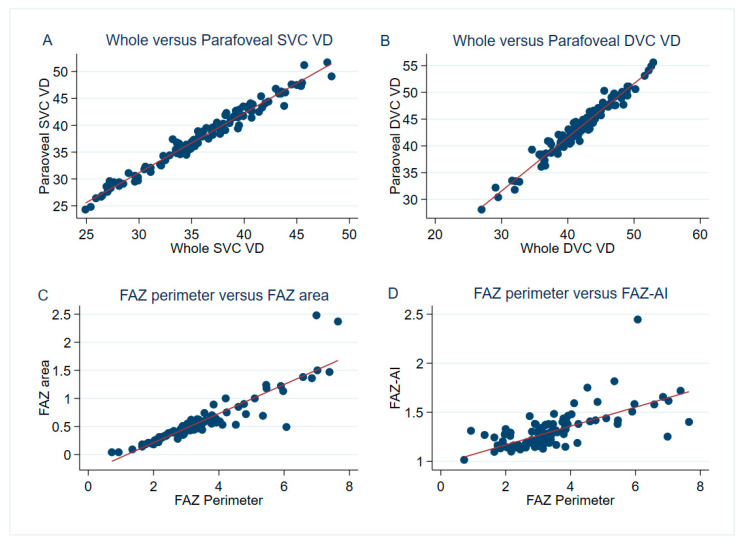
Representative pairs of optical coherence tomography angiography parameters with a significant correlation in the 3 × 3 mm scan area. The whole image superficial vascular complex (SVC) vessel density (VD) had an excellent correlation with the parafoveal SVC VD (*r* = 0.99, *p* < 0.001) (**A**). A very strong correlation was also found between the whole image deep vascular complex (DVC) VD and the parafoveal DVC VD (*r* = 0.98, *p* < 0.001) (**B**). There was a strong correlation between the foveal avascular zone (FAZ) perimeter and the FAZ area (*r* = 0.90, *p* < 0.01) (**C**), while the FAZ perimeter was positively correlated to the FAZ acircularity index (FAZ–AI) (*r* = 0.68, *p* < 0.001) (**D**).

The microvascular parameters examined are shown in Table 1 and Table 2 and Figure 2. Manual adjustment of the FAZ border was made in 13 eyes (3 for including extensive and continuous parafoveal capillary loss, 4 for redrawing the contour that initially encroached on macular exudation, and 6 for excluding vessels that were falsely included within the FAZ area). No manual adjustment of the segmentation was made. The mean FAZ area was 0.57 ± 0.38 mm^2^, which strongly correlated to the FAZ perimeter (mean 3.37 ± 1.30 mm, *r* = 0.90, *p* < 0.01). The 3 × 3 mm whole image DVC VD average was 41.81 ± 4.88%, which was strongly correlated to the parafoveal DVC VD (43.43 ± 5.01%, *r* = 0.98, *p* < 0.001). Notably, the DVC VD was usually more than the SVC VD regardless of the whole 3 × 3 mm image or the parafoveal region. The average 3 × 3 mm whole image SVC VD to DVC VD ratio was 0.87 ± 0.12, and the BCVA did not differ between eyes having a ratio of >1 and ≤1.

### 3.2. The Correlation between Visual Performance and OCTA Parameters

The BCVA correlated positively, ranging from weak to moderate, with the 3 × 3 mm whole image SVC VD, parafoveal SVC VD, 3 × 3 mm whole image DVC VD, parafoveal DVC VD, and FD–300 (Table 2). Among these measures, the BCVA correlated best with the 3 × 3 mm whole image DVC VD (*r* = 0.43, *p* < 0.001), indicating a better correlation than the whole image SVC VD (*r* = 0.30, *p* < 0.01). Conversely, the FAZ area, FAZ perimeter, and FAZ–AI were the parameters that negatively correlated with the BCVA. Amid the three negative influencers, the FAZ area and perimeter affected the BCVA the most and equally (*r* = −0.33, *p* = 0.01). Similarly, the LLVA was better when the five VD mentioned increased and worse when the FAZ-associated parameters increased. In general, BCVA correlated better with the OCTA parameters than LLVA.

### 3.3. Cutoff Points for Identifying Visual Impairment 

We assessed whether there is a threshold of OCTA parameters beyond which the probability of mild visual impairment increases. Mild visual impairment was defined as <70 ETDRS letters for both BCVA and LLVA.

**Figure 3:**
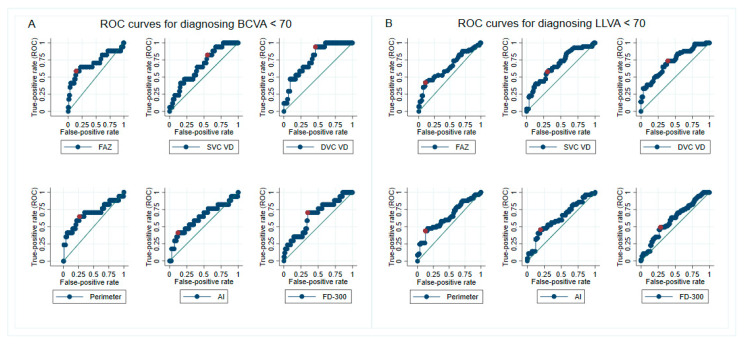
Receiver operating characteristic (ROC) curves of different parameters in identifying mild visual impairment. The cutoff point (marked as a red-filled circle) was defined as the threshold having the largest sum of sensitivity and specificity in determining visual impairment in daylight (**A**) or dim light (**B**). Special attention can be drawn to the 41.9% whole image deep vascular complex vessel density (DVC VD) (Top row, third from the left), which had the best discriminatory ability to diagnose patients with best-corrected visual acuity (BCVA) < 70 Early Treatment Diabetic Retinopathy Study (ETDRS) letters (area under the curve = 0.77, sensitivity 94%, specificity 54%, likelihood ratio = 2.04). Abbreviations: AI = acircularity index; BCVA = best-corrected visual acuity; DVC = deep vascular complex; FAZ = foveal avascular zone; FD–300 = parafoveal 300-µm ring vessel density; LLVA = low-luminance visual acuity; ROC = receiver operating characteristic; SVC = superficial vascular complex; VD = vessel density.

Receiver operating characteristic (ROC) curves were generated to find the appropriate cutoff capable of diagnosing mild visual impairment for all the microvasculature parameters (Figure 3). For BCVA, the 3 × 3 mm whole image DVC VD had the largest area under the curve (AUC) = 0.772 (Figure 3A and Table 3). A whole image DVC VD ≤ 41.9% had 94% sensitivity to diagnose BCVA < 70 ETDRS letters (positive post-test likelihood ratio [LR+] = 2.04), while a whole image DVC VD > 41.9% had 54% specificity to confirm BCVA ≥ 70 ETDRS letters. The pattern of distribution by utilizing 41.9% whole image DVC VD to discriminate between BCVA < 70 and BCVA ≥ 70 ETDRS letters was not arbitrary (*p* = 0.004) (Table 4), further implying that there was a considerable probability of differentiating impaired vision from good vision by setting 41.9% whole image DVC VD as a cutoff. A post-test LR of 2 was also of modest significance, increasing the disease probability by 15% [26].

The 3 × 3 mm whole image DVC VD also had the best accuracy (AUC = 0.719) in determining visual impairment under mesopic conditions (Figure 3B and Table 3). The sensitivity and the specificity were 74% and 61%, respectively, for discriminating between LLVA < 70 and ≥ 70 ETDRS letters by applying a 3 × 3 mm whole image DVC VD of 42.5% (post-test LR+ = 1.90). Moreover, the subclassification using LLVA 70 letters and DVC VD 42.5% was helpful as it did not appear by chance (*p* = 0.001) (Table 4).

Although the FAZ area did not yield the largest AUC, it had the best post-test LR to identify visual impairment (4.21 for BCVA and 3.82 for LLVA), which will increase the disease probability by approximately 25% [26]. The result was attributed to the high specificity (0.86 and 0.89 for BCVA and LLVA, respectively) when appointing the FAZ area as a discriminatory factor (cutoff at 0.64 mm^2^ for BCVA and 0.60 mm^2^ for LLVA).

When applying the most sensitive OCTA parameter to select eligible patients from our cohort, 61 out of 115 (53%) eyes will be shortlisted for having a whole image DVC VD ≤ 42%. Of these, only 26% (16/61) with BCVA < 70 letters or 64% (39/61) with LLVA < 70 letters may be considered eligible for trials. The positive predictive rate of applying one parameter to recruit the ideal trial participants would be too low.

**Figure 4:**
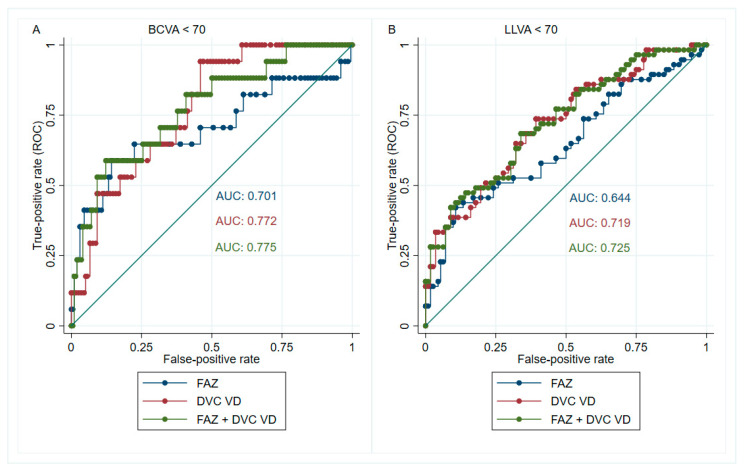
The receiver operating characteristic (ROC) curves for combined parameters in identifying visual impairment. The foveal avascular zone (FAZ) area and the whole image deep vascular complex vessel density (DVC VD) were combined to increase the diagnostic accuracy of visual impairment in photopic (**A**) and mesopic (**B**) conditions. The area under the curve (AUC) was 0.775 for diagnosing best-corrected visual acuity (BCVA) < 70 letters (**A**); the AUC was 0.725 for diagnosing low-luminance visual acuity (LLVA) < 70 letters (**B**).

However, if two OCTA parameters are applied together, the positive predictive rate will increase substantially. For example, 45% (10/22) of the eyes possessing both FAZ area ≥ 0.64 mm^2^ and whole image DVC VD ≤ 41.9% would have BCVA < 70 letters. Likewise, 77% (20/26) of the eyes with FAZ ≥ 0.6 mm^2^ and whole image DVC VD ≤ 42.5% would have LLVA < 70 letters, showing early impairment of mesopic vision. Indeed, combining the FAZ area and whole image DVC VD yielded an AUC of 0.775 (95% confidence interval (CI) = 0.636 to 0.890) for discriminating between BCVA < 70 and BCVA ≥ 70. Likewise, the combined AUC was 0.725 (95% CI = 0.621 to 0.813) for differentiating between LLVA < 70 and LLVA ≥ 70 (Figure 4).

## 4. Discussion

Our main finding is that the whole image DVC VD had the best discriminatory ability to detect mild visual impairment in DMI, with a cutoff point at 41.9% for BCVA and 42.5% for LLVA. The results indicate the potential utility of 3 × 3 mm whole image DVC VD at 42% to define vision-threatening DMI. It will also allow an enriched study cohort who are most likely to deteriorate functionally. Moreover, when considering eligibility criteria for clinical trials on interventions for DMI, it will be helpful to stratify patients based on whole image DVC VD less than 42% or not. In that way, the outcome of the pre-specified subgroups can be analyzed and compared.

When we applied the cutoff of whole image DVC VD 42% to estimate the prevalence of vision-threatening DMI in stable PDR eyes with BCVA ≥ 40 letters, 53% of the study population met this definition. However, only 26% of them would have BCVA < 70 letters. On the contrary, 64% would have LLVA < 70 letters. Therefore, BCVA may be a better outcome measure for assessing the prevention of further visual function loss, while LLVA may be better at evaluating the improvement of visual function in future interventions for DMI.

On the other hand, although the FAZ area did not yield the largest AUC, it had the best post-test LR+ in determining visual impairment in DMI (the cutoff for BCVA < 70 letters and LLVA < 70 letters was 0.64 mm^2^ and 0.60 mm^2^, respectively). Consequently, when we combined both cutoffs of the FAZ area and whole image DVC VD to identify vision-threatening DMI, the accuracy improved enormously (AUC = 0.775 and 0.725 for diagnosing BCVA < 70 letters and LLVA < 70 letters, respectively).

One interesting finding was that the perimeter of FAZ only had a weak correlation to all the VD parameters. Therefore, focal capillary losses in the margin of the foveal avascular zone do not seem to reflect the disease state in the 3 × 3 mm scan area. We suggest that changes in the FAZ area and the 3 × 3 mm whole image VD complement each other, and the combination of these two is better for identifying people with vision-threatening DMI.

We also observed that the 3 × 3 mm whole image VD of SVC and DVC correlated with the parafoveal VD of SVC and DVC, respectively. This finding suggests that DMI affects the whole 3 × 3 mm area, and measuring the therapeutic effect on the 3 × 3 mm whole image VD may be better than the parafoveal VD only.

When we consider the correlation between the microvascular parameters and the visual function, the 3 × 3 mm whole image DVC VD had the best correlation with BCVA and LLVA. All other parameters, including the FAZ area, showed a weak correlation with vision. Similar to our findings, Samara et al. reported a better correlation with logMAR VA in the DVC VD (*ρ* = –0.50, *p* < 0.001) than the superficial FAZ area (*ρ* = 0.29, *p* < 0.01) across various DR severity levels [27].

It is known that LLVA reflects both cone and rod functions [11]. Besides, eyes with LLVA < 70 letters were more prevalent than those with BCVA < 70 letters in our DMI study. Therefore, we further examined the relationship between LLVA and OCTA parameters. Several reports have shown that rod-induced hypoxia during dark adaptation may contribute to the pathogenesis of diabetic retinopathy [28,29]. However, our analysis in this study replicated a previous study that showed a strong correlation between BCVA and LLVA in PDR, suggesting that LLVA, like BCVA, is predominantly a cone function [12]. In addition, a previous report demonstrated that lower DVC VD was associated with reduced cone density (*r* = 0.43, *p* = 0.028) [30]. Together, these observations suggest that cones may be as vulnerable to metabolic alterations of diabetes at the macula as rods [31].

Since low-luminance deficiency (LLD) has been proposed as a reliable predictor of visual prognosis in age-related macular degeneration [32,33], we also analyzed the relationship between LLD and different OCTA parameters, which showed no correlation. The explanation could be that LLD is complex in interpretation, involving baseline VA, dark-adapted cone function, and eccentric fixation development [33].

There are several strengths of the present study. First, we identified the most accurate microvascular parameters associated with visual impairment using cut-points rather than correlations. Second, we ensured a homogeneous population of stable PDR patients without DME while most studies evaluated the OCTA metrics from various DR levels [34,35,36]. Third, the statistical methods employed in this study, namely the generalized estimating equations (GEE) model and bias-corrected bootstrapping strategy, corrected the inter-eye correlation [22,23]. Therefore, the estimated results in our report would be more precise at determining vision-threatening DMI than the current DMI definition based on FAZ size only.

Our study has some limitations. First, we only included stable-treated PDR patients, and therefore the results may not be extrapolated to other stages of DR. However, DMI in lower severity levels of DR is less prevalent. Second, this study focused on OCTA metrics only. Our further work will evaluate the role of OCT markers, such as disorganization of retinal inner layers or ellipsoid zone loss that may provide more information on visual outcomes [37,38,39]. It is unclear whether the microvascular findings on OCTA are the causes or the consequences of these OCT structural alterations [37,40,41]. It would be prudent to interrogate the course of these events in a longitudinal study [42]. Finally, the findings from this single-centered study need to be replicated. The cross-sectional nature of this study can only ascertain associations and limits its use in predicting progression. Nevertheless, the results provide valuable information on designing future clinical trials to prevent the progression of visual impairment in DMI.

## 5. Conclusions

In conclusion, our study has identified that combining cutoffs of the FAZ area and the whole image DVC VD in the 3 × 3 mm foveal center may be worth considering when defining vision-threatening DMI, and the whole image DVC VD had the best discriminatory ability among all the OCTA parameters to screen DMI-related mild visual impairment.

## Figures and Tables

**Table 1:** Baseline systemic and ocular characteristics.

**Demographics**	**Results**	**N**
**PATIENTS**		87
Age (years) (mean ± SD)	56.1 ± 12.5	87
>50 years	70.1%	61
Males	59.8%	52
Females	40.2%	35
T1DM	37%	32
T2DM	63%	55
Duration of diabetes (years) (mean ± SD)	27.1 ± 13.2	78
Bilateral eyes	52.9%	46
**Ocular Characteristics**	**Results**	**N**
**EYES**		123
BCVA (ETDRS Letters) (mean ± SD)	76 ± 10	123
≥70 letters	82.1%	101
<70 letters	17.9%	22
LLVA (ETDRS Letters) (mean ± SD)	66 ± 12	121
≥70 letters	47.1%	57
<70 letters	52.9%	64
LLD (ETDRS Letters) (median [IQR])	10 [6,7,8,9,10,11,12,13]	121
≥10 letters	48.8%	62
<10 letters	51.2%	59
Pseudophakia	47.2%	58
**OCTA (3 × 3 mm scan)**	**Results**	**N ^a^**
Image quality	7 ± 1	115
FAZ area (mm^2^)	0.57 ± 0.38	115
FAZ perimeter (mm)	3.37 ± 1.30	115
FAZ–AI	1.30 ± 0.18	115
Whole image SVC VD (%)	36.29 ± 5.31	115
Whole image DVC VD (%)	41.81 ± 4.88	115
Parafoveal SVC VD (%)	38.15 ± 5.97	115
Parafoveal DVC VD (%)	43.43 ± 5.01	115
FD–300 (%)	43.61 ± 4.92	115
Whole image SVC VD/DVC VD ratio	0.87 ± 0.12	115
SVC VD/DVC VD > 1.0	16.5%	19
SVC VD/DVC VD ≤ 1.0	83.5%	96

^a^ Only those 3 × 3 mm OCTA scans with a quality of 5 or more out of 10 were included for analysis. Abbreviations: AI = acircularity index; BCVA = best-corrected visual acuity; DVC = deep vascular complex; ETDRS = Early Treatment Diabetic Retinopathy Study; FAZ = foveal avascular zone; FD–300 = parafoveal 300-µm ring vessel density; IQR = interquartile range; LLD = low-luminance deficiency; LLVA = low-luminance visual acuity; OCTA = optical coherence tomography angiography; SVC = superficial vascular complex; SD = standard deviation; T1DM = type 1 diabetes mellitus; T2DM = type 2 diabetes mellitus; VD = vessel density.

**Table 2:** The correlation between different optical coherence tomography angiography parameters in the 3 × 3 mm scan area.

Pearson Correlation	BCVA	LLVA	FAZ Area	FAZ Perimeter	FAZ–AI	wi SVC VD	wi DVC VD	para SVC VD	para DVC VD	FD–300
BCVA	1.00									
LLVA	0.79 ***	1.00								
FAZ area	−0.33 **	–0.27 **	1.00							
FAZ perimeter	–0.33 **	–0.30 **	0.90 **							
FAZ–AI	–0.18	–0.20 *	0.35 *	0.68 ***	1.00					
wi SVC VD	0.30 **	0.34 **	–0.32 ***	–0.32 **	–0.09	1.00				
wi DVC VD	0.43 ***	0.42 ***	–0.48 ***	–0.45 ***	–0.13	0.50 ***	1.00			
para SVC VD	0.29 **	0.31 ***	–0.29 **	–0.27 **	–0.07	0.99 ***	0.47 ***	1.00		
para DVC VD	0.39 ***	0.38 ***	–0.43 ***	–0.37 ***	–0.07	0.46 ***	0.98 ***	0.44 ***	1.00	
FD–300	0.27 **	0.23 *	0.12	0.15	0.18	0.54 ***	0.48 ***	0.54 ***	0.50 ***	1.00

Abbreviations: AI = acircularity index; BCVA = best-corrected visual acuity; FAZ = foveal avascular zone; FD–300 = parafoveal 300-µm ring vessel density; LLVA = low-luminance visual acuity; para DVC VD = parafoveal deep vascular complex vessel density; para SVC VD = parafoveal superficial vascular complex vessel density; wi DVC VD = whole image deep vascular complex vessel density; wi SVC VD = whole image superficial vascular complex vessel density. *p*-values were reported from the F-test with standard errors clustered on each patient. *** *p* < 0.001, ** *p* < 0.01, * *p* < 0.05.

**Table 3:** The diagnostic values of different parameters in identifying visual impairment.

**For Diagnosing BCVA < 70 ETDRS Letters**							
**Parameters**	**AUC (95% CI) ^a^**	**Cutoff Point**	**Sensitivity**	**Specificity**	**PPV**	**NPV**	**LR+**
FAZ area	0.701 (0.525–0.865)	≥0.64 mm^2^	0.59	0.86	0.42	0.92	4.21
FAZ perimeter	0.684 (0.516–0.846)	≥3.49 mm	0.65	0.73	0.30	0.92	2.41
FAZ–AI	0.630 (0.451–0.785)	≥1.42	0.35	0.88	0.35	0.89	2.92
Whole image SVC VD	0.666 (0.528–0.797)	≤37.70%	0.82	0.44	0.20	0.93	1.46
Whole image DVC VD	0.772 (0.657–0.872)	≤41.9%	0.94	0.54	0.26	0.98	2.04
FD–300	0.661 (0.508–0.804)	≤42.82%	0.71	0.65	0.26	0.93	2.03
**For Diagnosing LLVA < 70 ETDRS Letters**							
**Parameters**	**AUC (95% CI) ^a^**	**Cutoff Point**	**Sensitivity**	**Specificity**	**PPV**	**NPV**	**LR+**
FAZ area	0.644 (0.544–0.744)	≥0.60 mm^2^	0.42	0.89	0.80	0.60	3.82
FAZ perimeter	0.642 (0.534–0.741)	≥3.59 mm	0.44	0.88	0.78	0.60	3.67
FAZ–AI	0.619 (0.514–0.732)	≥1.33	0.42	0.80	0.69	0.58	2.10
Whole image SVC VD	0.676 (0.557–0.770)	≤35.8%	0.58	0.70	0.66	0.62	1.93
Whole image DVC VD	0.719 (0.616–0.804)	≤42.5%	0.74	0.61	0.66	0.69	1.90
FD–300	0.601 (0.500–0.700)	≤42.82%	0.49	0.71	0.64	0.58	1.69

^a^ 1000 bootstrap replications were used to generate the bias-corrected 95% confidence intervals for the receiver operating characteristic (ROC) area under the curve (AUC). Abbreviations: AI = acircularity index; AUC = area under the curve; BCVA = best-corrected visual acuity; CI = confidence interval; DVC = deep vascular complex; ETDRS = Early Treatment Diabetic Retinopathy Study; FAZ = foveal avascular zone; FD–300 = parafoveal 300-µm ring vessel density; LLVA = low-luminance visual acuity; LR+ = positive likelihood ratio; NPV = negative predictive value; PPV = positive predictive value; ROC = receiver operating characteristic; SVC = superficial vascular complex; VD = vessel density.

**Table 4:** The pattern of distribution by applying different cutoff points.

Cutoff	BCVA < 70 (n)	BCVA ≥ 70 (n)	GEE *p*-value ^a^	Cutoff	LLVA < 70 (n)	LLVA ≥ 70 (n)	GEE *p*-Value ^a^
FAZ area ≥ 0.64 mm^2^	10	14	<0.001	FAZ area ≥ 0.6 mm^2^	24	6	0.001
FAZ area < 0.64 mm^2^	7	84		FAZ area < 0.6 mm^2^	33	50	
Perimeter ≥ 3.49 mm	11	26	0.002	Perimeter ≥ 3.59 mm	25	7	0.002
Perimeter < 3.49 mm	6	72		Perimeter < 3.59 mm	32	49	
FAZ–AI ≥ 1.42	6	11	0.010	FAZ–AI ≥ 1.33	24	11	0.012
FAZ–AI < 1.42	11	87		FAZ–AI < 1.33	33	45	
wi SVC VD > 37.7%	3	43	0.051	wi SVC VD > 35.8%	24	39	0.003
wi SVC VD ≤ 37.7%	14	55		wi SVC VD ≤ 35.8%	33	17	
wi DVC VD > 41.9%	1	53	0.004	wi DVC VD > 42.5%	15	34	0.001
wi DVC VD ≤ 41.9%	16	45		wi DVC VD ≤ 42.5%	42	22	
FD–300 > 42.82%	5	64	0.007	FD–300 > 42.82%	29	40	0.017
FD–300 ≤ 42.82%	12	34		FD–300 ≤ 42.82%	28	16	

^a^ Generalized estimating equation (GEE) model was used to specify the robust *p*-values in an unstructured correlation matrix. Abbreviations: AI = acircularity index; BCVA = best-corrected visual acuity; GEE = generalized estimating equations; FAZ = foveal avascular zone; FD–300 = parafoveal 300-µm ring vessel density; LLVA = low-luminance visual acuity; wi DVC VD = whole image deep vascular complex vessel density; wi SVC VD = whole image superficial vascular complex vessel density.

## Data Availability

The data presented in this study are available on request from the corresponding author. The data are not publicly available due to patients’ confidentiality.
